# *Culicoides* Latreille in the sun: faunistic inventory of *Culicoides* species (Diptera: Ceratopogonidae) in Mayotte (Comoros Archipelago, Indian Ocean)

**DOI:** 10.1186/s13071-019-3379-x

**Published:** 2019-03-22

**Authors:** Claire Garros, Karien Labuschagne, Laure Dommergues, M’sa Ben, Thomas Balenghien, Facundo Muñoz, Mame Thierno Bakhoum, Eric Cardinale, Hélène Guis

**Affiliations:** 1 Cirad, UMR ASTRE, 97490 Ste Clotilde, La Réunion France; 2 ASTRE, Univ Montpellier, Cirad, INRA, Montpellier, France; 3 Onderstepoort Veterinary Research, Agricultural Research Council-Onderstepoort Veterinary Research, EPV, Onderstepoort, South Africa; 4 Coopadem, Ouangani, Mayotte; 5 Cirad, UMR ASTRE, Rabat, Morocco; 6 IAV Hassan II, MIMC, Rabat, Morocco; 7 Cirad, UMR ASTRE, 34398 Montpellier, France; 8 Cirad, UMR ASTRE, Antananarivo, Madagascar; 9 Epidemiology and Clinical Research Unit, Institut Pasteur de Madagascar, Antananarivo, Madagascar; 10 FOFIFA DRZVP, Antananarivo, Madagascar

**Keywords:** *Culicoides*, Diversity, Spatial distribution, Afrotropical region, Mayotte

## Abstract

**Background:**

The south-west insular territories of the Indian Ocean have recently received attention concerning the diversity of arthropods of medical or veterinary interest. While a recent study highlighted the circulation of *Culicoides*-borne viruses, namely bluetongue and epizootic hemorrhagic disease, with clinical cases in Mayotte (comprising two islands, Petite-Terre and Grand-Terre), Comoros Archipelago, no data have been published concerning the species diversity of *Culicoides* present on the two islands.

**Results:**

A total of 194,734 biting midges were collected in 18 sites, covering two collection sessions (April and June) in Mayotte. Our study reports for the first time livestock-associated *Culicoides* species and recorded at least 17 described Afrotropical species and one undescribed species. The most abundant species during the April collection session were *C. trifasciellus* (84.1%), *C. bolitinos* (5.4%), *C. enderleini* (3.9%), *C. leucostictus* (3.3%) and *C. rhizophorensis* (2.1%). All other species including *C. imicola* represented less than 1% of the total collection. Abundance ranged between 126–78,842 females with a mean and median abundance of 14,338 and 5111 individuals/night/site, respectively. During the June collection, the abundance per night was low, ranging between 6–475 individuals. Despite low abundance, *C. trifasciellus* and *C. bolitinos* were still the most abundant species. *Culicoides* sp. #50 is recorded for the first time outside South Africa.

**Conclusions:**

Our study reports for the first time the *Culicoides* species list for Mayotte, Comoros Archipelago, Indian Ocean. The low abundance and rare occurrence of *C. imicola*, which is usually considered the most abundant species in the Afrotropical region, is unexpected. The most abundant and frequent species is C. *trifasciellus*, which is not considered as a vector species so far, but its role needs further investigation. Further work is needed to describe *Culicoides* sp. #50 and to carry on faunistic investigations on the other islands of the archipelago as well as in neighboring countries.

**Electronic supplementary material:**

The online version of this article (10.1186/s13071-019-3379-x) contains supplementary material, which is available to authorized users.

## Background

The south-west insular territories of the Indian Ocean have recently received attention concerning the diversity of arthropods of medical or veterinary interest [1–9]. This has been motivated by the recent epidemiological situation in the area. Even though the malaria morbidity and mortality has declined on some islands [10, 11], the region has faced major outbreaks of chikungunya [12–15] and endemic circulation of dengue virus [16, 17], West Nile virus [18, 19], Rift valley fever virus [20–26], bluetongue virus (BTV) and epizootic hemorrhagic disease virus (EHDV) [27–30] among others.

*Culicoides* are small biting midges (Diptera: Ceratopogonidae) distributed worldwide and implicated in the transmission of important viruses to ruminants (BTV, EHDV, Akabane virus), and equids (African horse sickness virus, AHSV) [31, 32]. The study of Afrotropical fauna started long ago [33–40] and recent work (starting in the 1990s) has tremendously updated these records [7, 41–51]. To date, the number of *Culicoides* species in the Afrotropical region is estimated to be around 190 species [50] with at least 120 species reported in the Southern African region [49].

In the south-west insular territories of the Indian Ocean, recent records mentioned five Afrotropical *Culicoides* species on La Reunion Island (*C. imicola*, *C. enderleini*, *C. bolitinos*, *C. grahamii* and *C. kibatiensis*) [7], where outbreaks of BTV and EHDV are regularly observed [30], and two Afrotropical species on Mauritius (*C. imicola* and *C. enderleini*) [52]. The faunistic inventory in Madagascar is probably largely incomplete as only 14 species have been recorded [48] and the precise identification of species related to *C. schultzei* needs further investigation [48, 53]. The Seychelles fauna for the genus *Culicoides* was investigated at three different times and three species were recorded (*C. leucosticus*, an Afrotropical species; *C. kusaiensis*, an Oriental species; and *C. adamskii*, reported only on a small Seychelles atoll) [54, 55]. Recent local reports highlighted the circulation of BTV and EHDV with some clinical cases in Mayotte, Comoros Archipelago [56]. Interestingly, no data are published concerning the *Culicoides* species present on this island.

Herein we report on a survey of *Culicoides* biting midges conducted in Mayotte, in the context of previous BTV and EHDV clinical cases [56]. A recent serosurvey on the island showed active circulation of both viruses throughout the island, with at least five BTV serotypes and one EHDV serotype [56]. Our survey is the first to address the *Culicoides* species diversity for the island and the whole archipelago. Our field survey covered different livestock breeding and production present on the island. Our specific objective was to describe the species diversity of *Culicoides* in Mayotte, to assess the abundance of dominant species and to map their spatial distribution to provide important insight to the epidemiology of the *Culicoides*-borne viruses on the island. Together with other published checklists for *Culicoides* in the region (South Africa, Kenya, La Réunion, Seychelles, Mauritius, Zimbabwe), we analyzed the species-area relationship (i.e. number of species in areas of different size irrespective of the identity of the species within the areas) to estimate the species richness in Madagascar.

## Methods

**Fig. 1:**
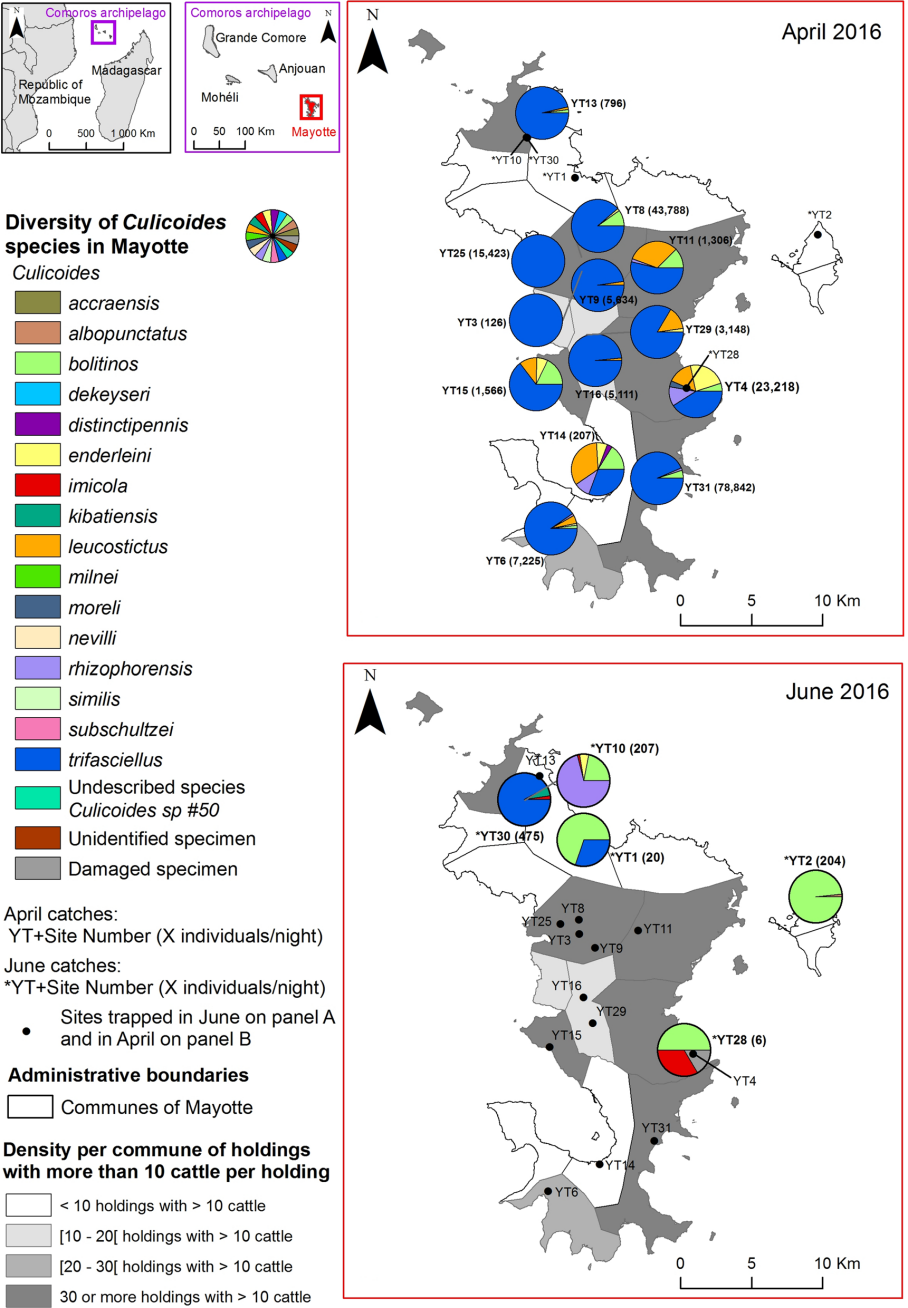
Diversity of *Culicoides* species recorded in Mayotte in two seasons, April 2016 and June 2016. The map was generated using ArcGIS 10.3 (ESRI). Administrative layers for Mayotte were extracted from Diva Gis (http://www.diva-gis.org/gData) and the GADM database (http://www.gadm.org, version 2.5, July 2015)

Mayotte is an overseas department of France in the south-west part of the Indian Ocean, located in the northern Mozambique Channel. The island, constituted of a main island (Grande-Terre) and a smaller one (Petite-Terre), belongs geographically to the Comoros Archipelago (Fig. 1). The soil type is mostly related to the volcanic origin of the island with massive soil erosion caused by heavy tropical rainfall on unprotected and deforested areas. The latest survey (2010) totaled 5700 cattle farms with 17,150 heads of cattle (less than 5 heads of cattle per farm on average), and 2200 sheep and goat farms with 12,600 animals (less than 6 animals per farm on average), highlighting the importance of smallholder farming. Breeding practices are mostly traditional with tethered cattle (72%) and small ruminants (51%) of local breeds. There is a single horse-riding center on Grande-Terre.

Thirteen sites were prospected, and collections were made from single night collections from 4th to 11th of April 2016, and 5 sites (YT1, YT2, YT10, YT28, YT30) from 20th to 27th of June 2016 (Fig. 1, see Additional file 1: Table S1). The timeframe in April was chosen to match the end of the rainy season when *Culicoides* populations are supposed to be the highest (dry season from April to November and rainy season from December to March). Selected sites were cattle (YT2, YT4, YT8, YT10, YT14, YT15, YT25, YT29, YT31), mixed farms with cattle and sheep and/or goats (YT1, YT6, YT11, YT13), sheep farms (YT9), goat farms (YT3, YT30) and the unique horse-riding center (YT28) to represent the different ruminants or equids present on the island. *Culicoides* trapping was done using a black light suction trap (Onderstepoort Veterinary Institute design, powered with a 12 V car battery) set up from before dusk until after sunrise, and positioned in the vicinity of the animal holdings (inside the shelter, along the fence, the closest tree for tethered animals) (Additional file 1: Table S1).

Specimens of *Culicoides* were stored in 70% alcohol until identification. Large samples were subsampled following a modified procedure described by Van Ark & Meiswinkel [57]. For each large sample, a 3 ml subsample was entirely sorted out and used to calculate the estimated total catch. All individuals were identified to the species level and sexed using a stereomicroscope. Morphological identification was performed using the available literature for the Afrotropical region [44, 49, 58, 59] and the expertise of KL. Biting midge specimens are deposited in the collection of Cirad, UMR ASTRE, Ste Clotilde, La Reunion, France (accession code: YT), and are available upon request to CG. Maps were generated using ArcGis^®^ software (version 10.3).

To determine the sampling efficiency at the end of the rainy season, species accumulation curves were plotted according to a randomization procedure using the R *vegan* package version 2.5-1 and by fixing the number of permutations to 100 [60]. A species accumulation curve derives as a plot of cumulative number of species discovered as a function of sampling effort. Each species is considered regardless of its abundance or rarity. The number of non-sampled species was extrapolated by estimating different richness indices (Chao, Chao bias-corrected, first order jackknife, second order jackknife and bootstrap estimators).

One of the reasons why islands are important in ecology and biogeography is that they are relatively isolated areas and therefore excellent natural laboratories to study the relationship between area and species diversity [61]. To estimate Madagascar’s *Culicoides* species diversity, we plotted the area-species curve of the south-west Indian Ocean using literature, our dataset for Mayotte and official island sizes. All analyses were performed using R (https://www.r-project.org) [62].

## Results

During the two collection sessions, 17 farms were prospected for 18 collection sites. Thirteen sites were prospected in April and 5 sites were prospected in June 2016. In one of the farms prospected in June, collections were carried out in two sites because of the presence of cattle (YT10) and goats (YT30) on two separate fields. The other sites prospected in June were the unique horse-riding center (YT28), one isolated goat and cattle farm in the north of Grande-Terre (YT1) and one site on Petite Terre (YT2) (Fig. 1).

**Fig. 2:**
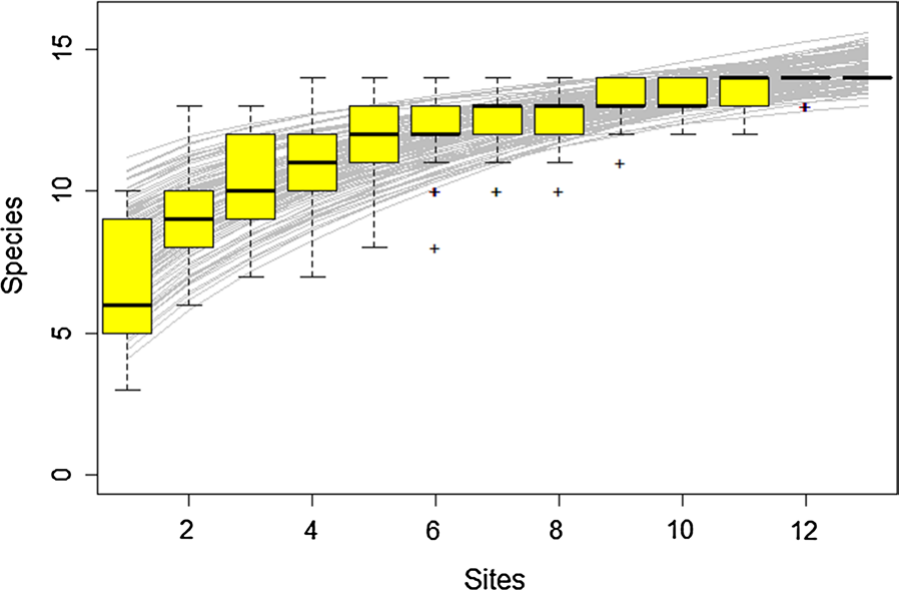
Species accumulation curve for the species observed in Mayotte. Boxplots mark standard deviations, crosses represent outlier points and grey curves represent the different simulations of richness indexes (Chao, Chao bias-corrected, first order jackknife, second order jackknife and bootstrap estimators)

At least 17 species were recorded during the two sessions (Table 1). One damaged specimen (absence of wings) collected in site YT28 was not identified. In site YT3, DNA of one specimen which could not be identified based on morphological features was extracted and the *cox*1 gene amplified. Unfortunately, the amplification failed. Out of the 17 species, 16 were known species distributed in the Afrotropical region (Table 2) and one was an undescribed species named *Culicoides* sp. #50 [49, 63, 64]. This latter species was collected in 9 sites (Fig. 1, Table 1). Ten species were collected both in April and in June: *Culicoides albopunctatus*, *C. bolitinos*, *C enderleini*, *C. imicola*, *C. leucostictus*, *C. rhizophorensis*, *C. similis*, *C. subschultzei*, *C. trifasciellus* and the undescribed species. Four species were collected only in April: *C. accraensis*, *C. distinctipennis*, *C. milnei* and *C. moreli.* Three species were only collected in June: *C. dekeyseri*, *C. kibatiensis* and *C. nevilli* plus one unidentified specimen (Fig. 1, Table 1). The species accumulation curve highlighted that the collection effort was sufficient to cover the species richness on Mayotte at the end of the rainy season (Fig. 2). Moreover, the different indices used to calculate extrapolated richness and compare it to our dataset showed that we could have missed from 1 (bootstrap) to 5 species (second order jackknife). Overall, this indicates that the inventory for the rainy season was notably robust and comprehensive.

**Table 1:** Number of *Culicoides* individuals (one-night collections) collected for the 18 sites. Thirteen sites were sampled from 3rd to 12th of April 2016, and 5 sites (YT1, YT2, YT10, YT28, YT30) from 20th to 27th of June 2016

Species	YT3	YT4	YT6	YT8	YT9	YT11	YT13	YT14	YT15	YT16	YT25	YT29	YT31	YT1	YT2	YT10	YT28	YT30	Positive sites	Total (%)
*C. trifasciellus*	122	9396	6451.5	38,160	5491	693	759	63.5	1000	4235	15,201	3066	72,191	6	1			431	16	157,267 (83.96)
*C. bolitinos*	1	1140	153	4240		160	18	33.5	276	12		6	3982	14	198	44	3		15	10,280.5 (5.49)
*C. enderleini*		5316	76.5	747	4		5	13.5	104	117	47	6	735		1	11		3	14	7186 (3.84)
*C. leucostictus*	1	3480	365.5	293	139	413	12	70	168	712	81	51	271					2	14	6058.5 (3.23)
*C. rhizophorensis*		2687	93.5			27		20	4				1044			143			7	4018.5 (2.15)
*C. moreli*		870		240							47								3	1157 (0.62)
*C. albopunctatus*			34				1		2	12			348		3				6	400 (0.21)
*C. imicola*		116		27					2				116		1	3	2	11	8	278 (0.15)
*C. distinctipennis*	1	174		27		13		6.5		23	12	6							8	262.5 (0.14)
Undescribed species *Culicoides* sp. #50			42.5	27			1		2		23	13	116			1		2	9	227.5 (0.12)
*C. subschultzei*			8.5						6		12		39			2			5	67.5 (0.04)
*C. milnei*		39																	1	39 (0.02)
*C. accraensis*				27															1	27 (0.01)
*C. kibatiensis*																		26	1	26 (0.01)
*C. similis*									2							1			2	3 (<0.01)
*C. dekeyseri*																1			1	1 (<0.01)
*C. nevilli*																1			1	1 (<0.01)
Unidentified specimen	1																		1	1 (<0.01)
Damaged specimen																	1		1	1 (<0.01)
No. of species	5	9	8	9	3	5	6	6	10	6	7	6	9	2	5	9	3	6		
Total	126	23,218	7225	43,788	5634	1306	796	207	1566	5111	15,423	3148	78,842	20	204	207	6	475		187,302

**Table 2:** Species list of *Culicoides* recorded in Mayotte, with systematic affiliations, published bionomics and current known distribution (review based on [49, 50])

Species	Systematic affiliation following Borkent’s catalogue	Systematic affiliation following [49]	Bionomics	Known distribution
*C. accraensis* Carter, Ingram & Macfie, 1920	Subgenus *Synhelea*	No subgenus affiliation known, Accraensis Group	Breeds primarily in tree holes, although there are several records from other habitat types. The host-feeding preference of *C. accraensis* is probably large mammals based on the low number of antennal sensilla. In Nigeria, adults were most numerous in the forest zone near livestock pens; abundance in relation to rainy season in Senegal.	Angola, Cameroon, Congo, Democratic Republic of Congo, Gambia, Ghana, Kenya, Nigeria, Senegal, Uganda, Zimbabwe
*C. albopunctatus* Clastrier, 1960	Subgenus *Synhelea*	No subgenus affiliation known, Accraensis Group	Tree-hole breeder species. In South Africa, rarely collected species and usually with low abundance.	Congo, Democratic Republic of Congo, South Africa, Tanzania
*C. bolitinos* Meiswinkel, 1989	Subgenus *Avaritia*	Subgenus *Avaritia*	Widespread distribution in South Africa. This species is described as breeding in the dung of cattle and buffaloes. In Zimbabwe, the species is abundant in regions characterized by low rainfall and high environmental temperatures, although in South Africa *C. bolitinos* has been reported to be more abundant in the cooler mountainous regions.	Botswana, Ivory Coast, Kenya, Lesotho, Malawi, Mauritius, Nigeria, Reunion, Senegal, South Africa, Zimbabwe
*C. dekeyseri* Clastrier, 1958	No subgenus affiliation known, Dekeyseri Group	No subgenus affiliation known, Dekeyseri Group	Not a common species in South Africa but apparently widespread in Nigeria in both forest and savanna areas.	Angola, Gambia, Mali, Nigeria, Senegal, South Africa, Uganda
*C. distinctipennis* Austen, 1912	Subgenus *Meijerehelea*	Subgenus *Meijerehelea*	Widespread and common in Nigeria. Recorded as primarily a bird-feeder but also collected feeding on humans in Zaire and Senegal. Breeding sites described as moist soil and mud taken from boggy ground, edges of pools or lakes, puddles and streams.	Angola, Cameroon, Congo, Egypt, Gambia, Ghana, Guinea, Kenya, Madagascar, Mali, Nigeria, Democratic Republic of Congo, Sao Tome, Senegal, Sierra Leone, South Africa, Sudan, Tanzania, Uganda, Zimbabwe
*C. enderleini* Cornet & Brunhes, 1994	Subgenus *Remmia*	Subgenus *Remmia*	Widespread and common species.	Ethiopia, Gambia, Kenya, Madagascar, Nigeria, Reunion Island, Senegal, South Africa, Tanzania, Uganda, Zimbabwe
*C. imicola* Kieffer, 1913	Subgenus *Avaritia*	Subgenus *Avaritia*	Very widely distributed and common species. Can be extremely abundant under ideal conditions. Historically recognized as the Afrotropical vector species of AHS and BT viruses.	Algeria, Angola, Burkina Faso, Cameroon, Chad, Congo, Democratic Republic of Congo, Egypt, Ethiopia, Gambia, Ghana, Guinea, Ivory Coast, Kenya, Madagascar, Mauritius, Morocco, Nigeria, Reunion, Sao Tome, Senegal, South Africa, Sudan, Tanzania, Uganda, Zimbabwe
*C. kibatiensis* Goetghebuer, 1935	Subgenus *Avaritia*	Subgenus *Avaritia*	Widespread and common species.	Kenya, Nigeria, Democratic Republic of Congo, Tanzania, Uganda
*C. leucostictus* Kieffer, 1911	Subgenus *Meijerehelea*	Subgenus *Meijerehelea*	–	Angola, Cameroon, Congo, Democratic Republic of Congo, Egypt, Ethiopia, Gambia, Ghana, Guinea, Kenya, Madagascar, Mali, Nigeria, Sao Tome, Senegal, Sierra Leone, South Africa, Sudan, Tanzania, Uganda, Zimbabwe
*C. milnei* Austen, 1909	Milnei Group	No subgenus affiliation known, Milnei Group	In South Africa, rarely collected species while widespread in the eastern part.	Democratic Republic of Congo, Madagascar, Nigeria, Senegal, South Africa, Uganda
*C. moreli* Clastrier, 1959	Milnei Group	No subgenus affiliation known, Milnei Group	Regularly collected in high numbers from KwaZulu-Natal in South Africa while rare elsewhere in the country.	Gambia, Ivory Coast, Kenya, Madagascar, Nigeria, Senegal, South Africa, Sudan, Uganda
*C. nevilli* Cornet & Brunhes, 1994	Subgenus *Remmia*	Subgenus *Remmia*	Widespread and common species in South Africa.	Democratic Republic of Congo, Kenya, Madagascar, Nigeria, Senegal, South Africa, Zimbabwe
*C. rhizophorensis* Khamala & Kettle, 1971	Subgenus *Remmia*	Subgenus *Remmia*	Rare species, collected along the eastern and southern coastal area near swampy and saline areas in South Africa.	Comoros Islands, Madagascar, Kenya, South Africa
*C. similis* Carter, Ingram & Macfie, 1920	Subgenus *Synhelea*	No subgenus affiliation known, Similis Group	Widespread and common in the Afrotropical region, abundant in Senegal during the dry season, breeding sites in mud from the edges of temporary pools, puddles or ponds, and rotting banana stems or other vegetation	Burkina Faso, Egypt, Ethiopia, Gambia, Ghana, Kenya, Mali, Nigeria, Senegal, South Africa, Sudan, Tanzania, Zimbabwe
*C. subschultzei* Cornet & Brunhes, 1994	Subgenus *Remmia*	Subgenus *Remmia*	Widespread and common species. Could be locally abundant.	Ethiopia, Kenya, Senegal, South Africa, Zimbabwe
*C. trifasciellus* Goetghebuer, 1935	Subgenus *Avaritia*	Subgenus *Avaritia*	In Kenya, species collected from high altitude forest and grass-land and reported as major human biting species. Reported primarily as diurnal with peaks in morning hours and in the late evening.	Democratic Republic of Congo, Kenya, Senegal, Tanzania, South Africa

A total of 194,734 individuals were collected in the 18 sites during 20 collection nights (Table 1). Indeed, due to social protests resulting in road barricades, two sites (YT6 and YT14) could not be reached on the morning after the trap was set up but only the day after. As the lights and fans of these two traps were still running correctly, we assumed that the mean of the total catch of the two nights of each trap best represented single night catch estimates. Overall, 98.29% of individuals were females (191,401) and 1.71% were males (3333). Taking into account 18 collection nights, a total of 187,302 individuals were identified during the two sessions (mean and median catch per night 10,406 and 1436, respectively) with 98.25% of females (184,026.5) and 1.75% of males (3275.5).

As expected, over 99.5% of the individuals (186,390 individuals) were caught in the 13 sites sampled in April and only 912 individuals were collected in June. Percentage of females in April was 98.26 in June (183,137.5 females) and 97.48 in April (889 females).

**Fig. 3:**
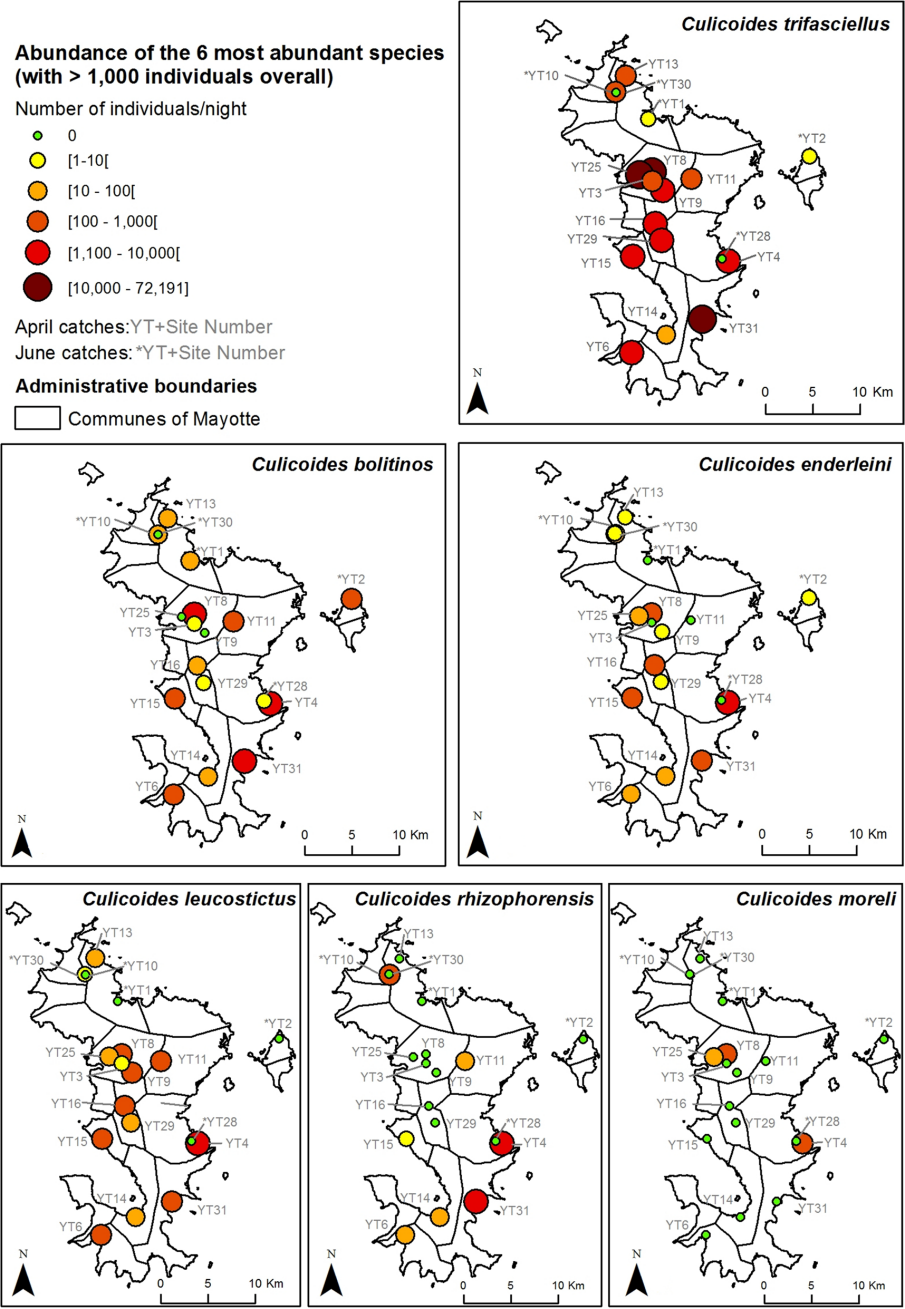
Abundance maps for the six most abundant species. The map was generated using ArcGIS 10.3 (ESRI). Administrative layers for Mayotte were extracted from Diva Gis (http://www.diva-gis.org/gData) and the GADM database (http://www.gadm.org, version 2.5, July 2015)

**Fig. 4:**
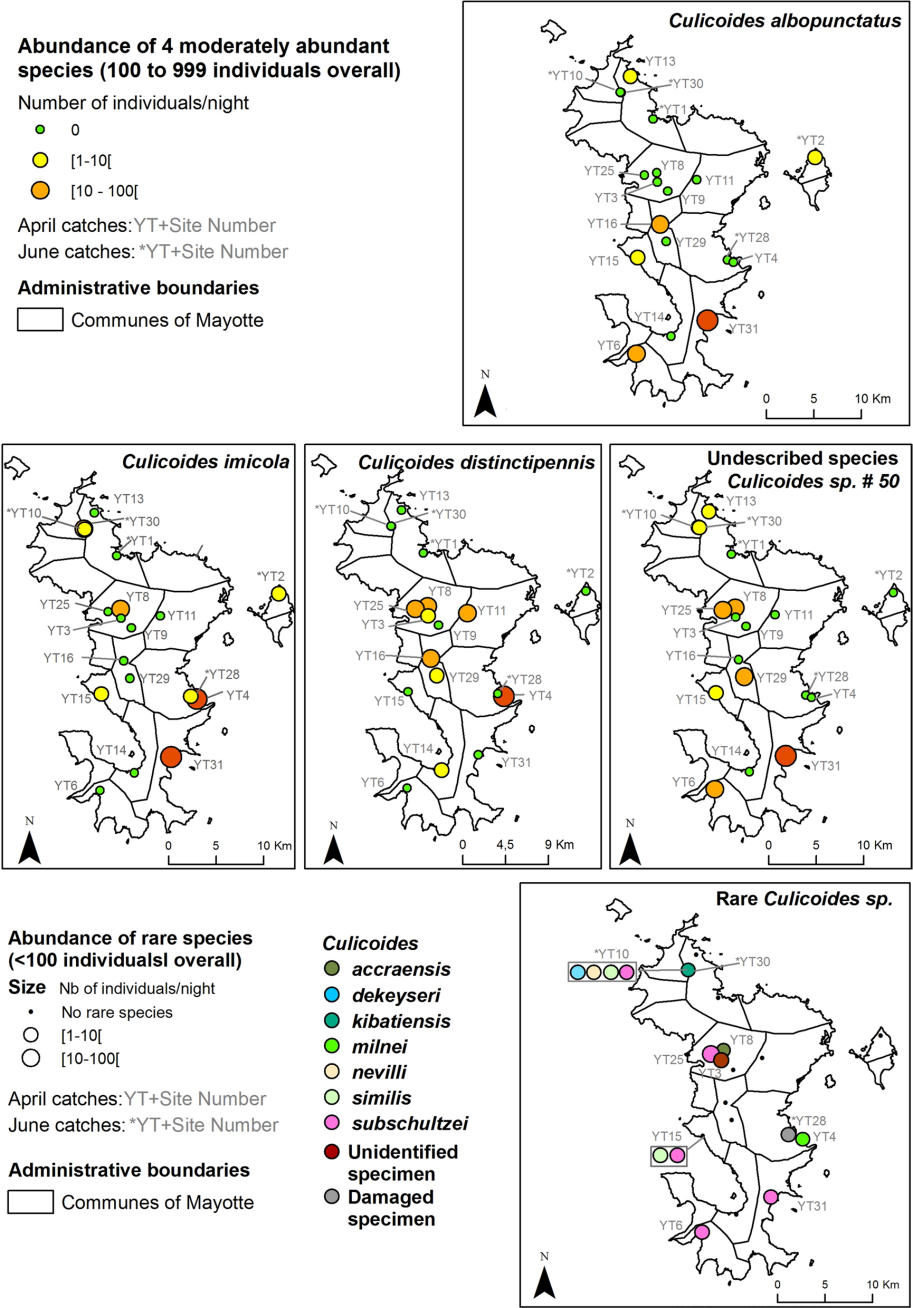
Abundance maps for the species with moderate and rare occurrence. The map was generated using ArcGIS 10.3 (ESRI). Administrative layers for Mayotte were extracted from Diva Gis (http://www.diva-gis.org/gData) and the GADM database (http://www.gadm.org, version 2.5, July 2015)

Considering only the April collection session, the most abundant species were *C. trifasciellus* (84.1% of collection), *C. bolitinos* (5.4%), *C. enderleini* (3.9%), *C. leucostictus* (3.3%) and *C. rhizophorensis* (2.1%) (Figs. 1, 3). All other species including *C. imicola* represented less than 1% of catches (Table 1, Fig. 4). Three to ten species were collected per site (Table 1). Four sites (YT31, YT8, YT4 and YT25) represented 86.5% of the total catches. Abundance ranged from 126 to 78,842 females with a mean and median abundance of 14,338 and 5111 individuals/night/site, respectively. *Culicoides trifasciellus* was present in all 13 sites sampled in April and was the most abundant species in all but one site, YT14, where *C. leucostictus* was the most abundant (Table 1, Fig. 3).

During the June collection session, the abundance per night was low, ranging between 6 and 475 individuals. Mean and median abundance were 182 and 204 individuals/night/site, respectively. Despite low abundance, *C. trifasciellus* and *C. bolitinos* were still the most abundant species, representing 48.0 and 28.4% of catches, respectively; *C. rhizophorensis* represented 15.7% of catches and all other species represented less than 1% of catches (Table 1, Fig. 3). *Culicoides bolitinos* was the most abundant species in 3 out of the 5 sites; *C. rhizophorensis* and C. *trifasciellus* were the most abundant in YT10 and YT30, respectively. *Culicoides trifasciellus* was also present in 2 other sites but in low numbers (1 and 6 individuals in YT1 and YT2, respectively). The number of species collected per site varied between 2 and 9 (Fig. 1, Table 1).

**Fig. 5:**
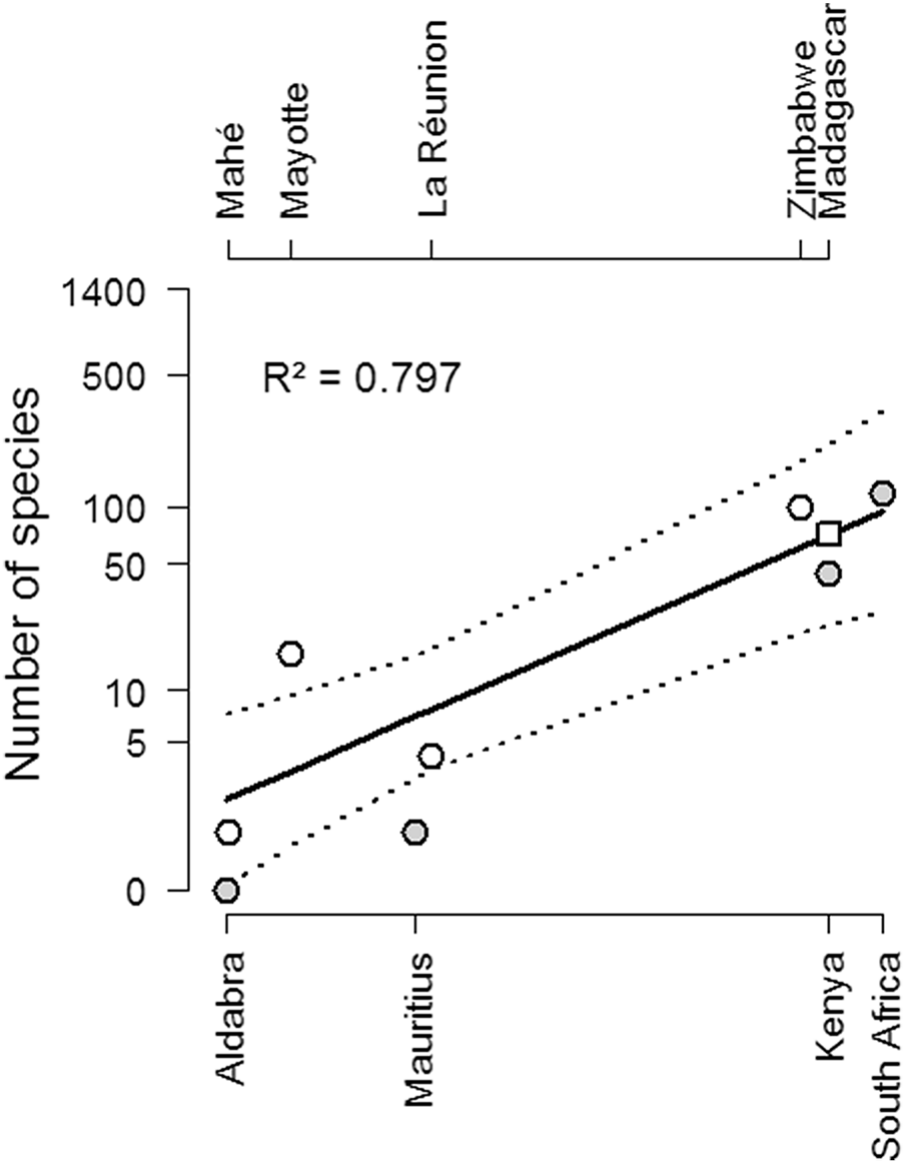
Species-area relationship in the south-west Indian Ocean region. Circle symbols represent the intersection of surface and the know number of species. Grey symbols refer to the lower X-axis. The square symbol represents the intersection of Madagascar’s surface and the regression line (black line, with 95% confidence interval in dashed lines). The X-axis represents the size of the island or country

The area-species curve was plotted using species lists previously published [7, 44, 47, 49, 52, 54, 55] and our dataset for Mayotte (Fig. 5). The correlation was relatively high (*R*^2^ = 0.797) and allowed to predict 71 species for Madagascar.

## Discussion

Faunistic inventory of mosquitoes was recently made in the south-western islands of the Indian Ocean [5, 6] but no such work has been done before for *Culicoides* species. Yet, completing a species checklist is the first fundamental step before any further epidemiological studies on vector species can be launched. Indeed, tropical oceanic islands are highly appropriate for surveys to attempt to complete a species checklist for a given group as they are isolated and endemism may be rampant [61].

Our study reports for the first time livestock-associated *Culicoides* species and records at least 17 described Afrotropical species with one undescribed species (*Culicoides* sp. #50). The reference trap for *Culicoides* collection was used because it can catch the highest diversity and abundance regardless of the season [65]. However, as it is a light-trap, species with diurnal activities may have been missed. Traps were localized in farms with cattle or small ruminants to investigate the species in close contact with hosts for BTV and EHDV. Therefore, we could have missed species breeding in other ecosystems such as sandy beaches, mangroves or sylvatic species. However, the species accumulative curve plot allows to consider our study as a good picture of the diversity of livestock-associated species at the supposed abundance peak (end of the rainy season). As the statistical analysis was carried out on species richness at the end of the rainy season, this work could be completed by another inventory during the dry season (only five sites were prospected in June).

Considering the size of Mayotte (374 km^2^) and its volcanic origin, the species diversity is high (Reunion island, five species for 2512 km^2^; Mauritius, two species for 1865 km^2^). All the species recorded in Mayotte had been previously found in South Africa [49], while the species presence and diversity composition was quite different from that recorded on neighboring island territories. In Mauritius, the last update inventory mentioned two species, *C. imicola* and *C. enderleini* [52], which needs to be confirmed with a large-scale survey. Different surveys recorded five species on La Réunion island [7, 66] and allow to consider this figure as robust. Unfortunately, the *Culicoides* diversity is clearly underestimated in Madagascar [53] and unknown on the other islands of the Comoros Archipelago. Our estimation of species number in Madagascar reached 71 species (Fig. 5). Although entomological surveys were undertaken in the Seychelles early in the past century [54], only two inventories were published for the islands. The oldest one [54] reported two species, *C. leucosticus*, an Afrotropical species, and *C. kusaiensis*, an Australasian species extending to Southeast Asia: Melanesia; Micronesia; Australia (Queensland) [67]; Thailand [68]; China [67]; and Malaysia [54]. This species list was further completed with a description of a new species from the Aldabra island, Seychelles, named *C. adamskii* [55]. All these collections in the different territories are incomplete and certainly require updating. The high species diversity recorded in Mayotte might be explained by the close geographical connection to the African continent which may facilitate *Culicoides* dispersal [69]. Indeed, the Comoros Archipelago has strong links to the African continent through current and past trade and human migrations.

*Culicoides imicola* was collected in eight sites and with a maximum abundance of 116 individuals per night. This is an unexpectedly low abundance and patchy distribution of *C. imicola*. *Culicoides imicola* is usually the most frequent and/or abundant species in the fringe of the African continent (Mediterranean basin and south of Africa where the climate is characterized by dry summers and rainy winters) [49, 65, 70, 71], while less abundant or frequent in other regions of the continent [42, 43, 72]. This could be related to soil type or other environmental variables. In our survey, this could be explained by the limited number of hosts and low cattle density and abundant sylvatic environment around the sites. The most abundant and frequent species was C. *trifasciellus.* This species has often been mentioned in Central Africa [37] or Kenya [73] studies and recently as a cave breeding species in Gabon [74]. It has been reported as an anthropophilic species [37, 49, 73] but no such nuisance was noticed during our fieldwork or reported by farmers. Moreover, *C. trifasciellus* is the vector species of *Onchocerca gutturosa*, a cattle microfilaria. We have no data attesting the presence of this parasite on the island, but it has been recorded on the African continent [75, 76].

To our knowledge and based on literature, there are no data on the vector role of *C. trifasciellus* for BTV or EHDV (Table 3). *Culicoides bolitinos* and *C. enderleini*, the 2nd and 3rd most abundant species respectively found in Mayotte, are known for their vector role [49]. The vector competence of *C. bolitinos* has been demonstrated in the laboratory for several viruses [77–80] and its host preference towards horses and ruminants has been documented [42, 58, 81, 82] which makes this species a major vector species for BTV, AHS and EHD virus in the Afrotropical region. *Culicoides enderleini* is highly suspected to be implicated in BTV transmission based on laboratory susceptibility studies and isolations of BTV in South African *Culicoides* populations [78, 80].

**Table 3:** Characterization of the epidemiological role of the species recorded in Mayotte based on [49, 50]

Species	Host-vector contact				Detection of viral genome	Detection of parasites	Vector competence in the laboratory
	Bovine	Equine	Sheep/goat	Other			
*C. accraensis*	+	Unknown	Unknown	Unknown	Unknown	Unknown	Unknown
*C. albopunctatus*	Unknown	Unknown	Unknown	Unknown	Unknown	Unknown	Unknown
*C. bolitinos*	+++	+++	+++	Unknown	BTV, AHSV, unidentified virus	Unknown	AHSV, BTV, EEV, EHDV
*C. dekeyseri*	Unknown	Unknown	Unknown	Unknown	Unknown	Unknown	Unknown
*C. distinctipennis*	Unknown	Unknown	Unknown	Birds, humans	Unknown	Unknown	Unknown
*C. enderleini*	+	+	+	Poultry	BTV	Unknown	
*C. imicola*	+++	+++	+++		AHSV, Akabane virus, BTV, BEDV, EEV, Letsitele virus, Nyabira virus, Sabo virus, Shamonda virus, Simbu virus, unidentified virus	Unknown	AHSV, BTV, EEV, EHDV
*C. kibatiensis*	Unknown	Unknown	Unknown	Unknown	Unknown	Unknown	Unknown
*C. leucostictus*	−	−	−	Birds, poultry	Unknown	Unknown	EHDV
*C. milnei*					Akabane virus, BTV	Unknown	
*C. moreli*					Unknown	Unknown	
*C. nevilli*	+	+	+	Poultry	EHDV	Unknown	
*C. rhizophorensis*					Unknown	Unknown	
*C. similis*					Unknown	Unknown	
*C. subschultzei*					Unknown	Unknown	
*C. trifasciellus*				Humans	Unknown	*Onchocerca gutturosa*	

*Key*: +, positive association; −, negative association

*Culicoides leucostictus* and *C. rhizophorensis* were frequently and abundantly collected in our survey. In a study by Venter et al. [78], BTV isolation was successful from one pool of *C. leucostictus* while it is a common and widespread species in South Africa, being dominant near birds [49, 83, 84]. Indeed, the species was not attracted by horse or sheep baits in a recent host vector contact study in Senegal [81]. Both species are reported to breed in swampy, saline areas and salt-marshes environment in South Africa, like those created by periodical flooding with sea water due to tidal activity [49, 83]. Indeed, *C. rhizophorensis* was notably collected in our survey in farms close to the coast.

*Culicoides* sp. #50 is reported for the first time outside its known distribution range, i.e. South Africa [49, 63]. This species was mentioned for the first time in the Kruger National Park in South Africa reared from the dung of elephant and plains zebras [64]. Our record updates the known species distribution and its biology as no big wild mammals are present in Mayotte.

The assumption that competence for orbiviruses might be widespread in the genus *Culicoides* encourages further assessment of the role of each species in relation to its abundance and seasonality [78]. Meanwhile, the potential involvement of numerous species in virus transmission, each exhibiting different bionomics and phenology, greatly increases the complexity of the epidemiology of *Culicoides*-borne viruses. Because of the limited number of livestock on the island and low ruminant density, species usually associated with livestock farming in the Afrotropical region were either collected in small numbers (C*. imicola*, *C. bolitinos* and *C. milnei*) or absent (*C. kingi*). We could also not exclude the assumption that local ecological conditions (soil composition) are not favorable for these species. The relatively large number of *C. leucostictus* and *C. rhizophorensis* might be due to the presence of natural larval habitats around the prospected farms. Overall, no clear spatial pattern was observed regarding species diversity or abundance.

*Culicoides* species delimitation is commonly known to be complicated by large morphological variations [85]. Recently, systematics and taxonomy of the Afrotropical species of *Culicoides* using molecular tools [41, 48, 86] or morphological characters [49] confirmed the existence of tentative undescribed new species for the region. Molecular data could provide more resolution of the species diversity collected in Mayotte. Furthermore, *C. trifasciellus* has a close undescribed taxa named *Culicoides* sp. #20 [86]. In light of these ongoing changes, one needs to be careful with the species list that reflects our taxonomic knowledge at the time of identification.

## Conclusions

Our study reports for the first time the *Culicoides* species list for Mayotte, Comoros Archipelago, Indian Ocean. Further work is needed to describe *Culicoides* sp. #50 and to carry on faunistic investigations on the other islands of the archipelago as well as in neighboring countries. The role of the most abundant species, *C. trifasciellus*, in the transmission of pathogens requires further investigation.

## Additional file

**Additional file 1: Table S1.** Description of study sites and trap localization.

## Abbreviations

BTV: bluetongue virus
EHDV: epizootic hemorrhagic disease virus
AHSV: African horse sickness virus
