# Supplementary material for: Culicoides Latreille in the sun: faunistic inventory of Culicoides species (Diptera: Ceratopogonidae) in Mayotte (Comoros Archipelago, Indian Ocean)
Source: Parasit Vectors. 2019 Mar 22;12:135. doi: 10.1186/s13071-019-3379-x (PMC6431056; doi:10.1186/s13071-019-3379-x)
Supplement: Supplementary file 1 — Additional file 1: Table S1. Description of study sites and trap localization. [file 13071_2019_3379_MOESM1_ESM.docx]

**Additional file 1: Table S1.** Description of study sites and trap localization.

| Site number | Site description and trap localization |
| --- | --- |
| YT1 | Muddy cattle farm, surrounded by tropical forest, around 70 cows and 60 goats, cattle outside in a fenced yard day and night  Trap set up in the cattle yard |
| YT2 | Trap set up in a tree close to 3 tethered cattle, semi-opened pasture |
| YT3 | Small goat shelter in the backyard of a house, 10 goats, shelter with limited openings  Trap set up outside the shelter, along the wall |
| YT4 | Cattle farm with several concrete feed stalls, 19 individuals wandering next to the farm during the day and on site in the morning for milking  Trap set up under one shelter, between the milking zone and a feed stall |
| YT6 | Stalling next to a road and surrounded by banana trees, 8 cows, presence of sheep (undetermined number)  Trap set up under the stalling |
| YT8 | Cattle farm with concrete feed stalls, numerous crops around the farm, 16 cows  Trap set up under the stalling |
| YT9 | Modern sheep farm, daily animal movements from the farm to a close pasture, sheep gathered in a semi-opened building at night, 42 individuals  Trap set up above the sheep park in the building |
| YT10 | Mixed farm with goats (undetermined number) and cattle (20 individuals)  Trap set up along the cattle stalling |
| YT11 | Wide open and concrete stalling, presence of 10 cows and 7 goats, animals outside in the pasture at day and in the stalling at night  Trap set up in the back of the stalling |
| YT13 | Mixed cattle and goat/sheep farm, cattle separated from goats/sheep, 15 cows and 20 sheep, 45 goats  Trap set up in the sheep/goat holding, building with limited openings |
| YT14 | Cattle farm close to a road and banana tree plantation, small shelter with wandering animals (9 cows)  Trap set up under the shelter |
| YT15 | Small cattle farm, with concrete feed stalls, 7 cows  Trap set up between two feed stalls |
| YT25 | Cattle farm, 8 individuals  Trap set up under the shelter |
| YT28 | Horse farm, horses in boxes all night (14 individuals)  Trap set up between two boxes |
| YT29 | Urban cattle farm with mud, 4 individuals outside in a fenced yard day and night  Trap set up along the yard wall |
| YT30 | Mixed farm with goats (undetermined number) and cattle (20 individuals)  Trap set up along the goat park fence |
| YT31 | Cattle farm with concrete feed stall, 9 individuals  Trap set up under the stalling |
